# Unveiling Anxiety Factors in Orthorexia Nervosa: A Qualitative Exploration of Fears and Coping Strategies

**DOI:** 10.3390/healthcare12090925

**Published:** 2024-04-30

**Authors:** Panagiota Tragantzopoulou, Vaitsa Giannouli

**Affiliations:** 1 School of Social Sciences, University of Westminster, 115 New Cavendish Street, London W1W 6UW, UK; 2 School of Medicine, Aristotle University of Thessaloniki, 54124 Thessaloniki, Greece; giannouliv@hotmail.com

**Keywords:** orthorexia nervosa, extreme healthy eating, anxiety, fears, coping strategies

## Abstract

Orthorexia nervosa represents a controversial phenomenon in the realm of eating practices, characterized by an obsessive fixation on consuming only foods deemed ’healthy’, and a preoccupation with food purity. While the existing literature has identified the presence of stressful behaviors among individuals with orthorexia, the precise factors and circumstances eliciting these stress-inducing emotions remain the subject of ongoing inquiry. This study aims to explore the triggers and situations that precipitate stressful beliefs and emotions among individuals who self-identify as preoccupied with healthy eating, as well as the coping mechanisms developed to manage these feelings. Through conducting one-to-one interviews with thirteen individuals, thematic analysis was employed to elucidate the nuances of their experiences. Participants articulated concerns regarding the contamination of foods available in supermarkets and restaurants, thereby prompting the meticulous selection of food sources and aversion to dining out. The prospect of trying new foods and engaging in social gatherings involving food emerged as anxiety-inducing scenarios, prompting individuals to adhere to monotonous dietary patterns and impose self-isolation. Additionally, medical appointments were perceived as stressful, driven by health concerns underpinning the adoption of stringent dietary practices. These findings underscore the spectrum of fears and coping mechanisms exhibited by individuals with orthorexia nervosa, which hold profound implications for their overall well-being.

## 1. Introduction

Orthorexia nervosa (ON) is recognized as a dietary pattern typified by a strict avoidance of foods perceived as ‘impure’, coupled with an intense fixation on adhering to a health-conscious diet [1,2]. It has also been proposed that ON is marked by a sense of superiority over individuals who consume foods deemed ‘unhealthy’ [3]. The primary motivation behind this fixation on healthy eating is often believed to stem from the desire to address physical health challenges, prevent future illnesses, and enhance overall health [3]. During the COVID-19 pandemic, for instance, the fear of contracting the virus and the desire to boost immunity were proposed as triggering factors in the escalation of individuals’ obsession with healthy eating [4]. More specifically, previous case studies examining individuals hospitalized for disordered eating have highlighted the emergence of ON-like behaviors following dietary modifications aimed at addressing health concerns. These modifications gradually evolved into an excessive fixation on healthy eating practices. Interestingly, despite observable weight loss, there was an absence of fear related to gaining weight [5,6,7].

A key difference between ON and anorexia is that the former presents an obsession with the quality of food whereas the latter presents a preoccupation with the quantity of the food [8,9]. Nevertheless, ON is believed to share similar characteristics to anorexia, including perfectionism and a strong inclination towards exerting control [10]. Although ON has not yet been officially recognized as a distinct eating disorder (ED), anecdotal reports suggest that the obsessive focus on food quality can lead individuals to eliminate entire food groups from their diet [11]. This can result in malnutrition, emotional distress, various health complications, and significant weight loss [12,13]. These findings underscore the significant ramifications of excessive adherence to extreme healthy eating patterns and restrictive dietary practices, which may initially stem from a commendable intention to mitigate prospective illnesses and safeguard personal well-being. However, such behaviors ultimately may pose a threat to health integrity.

Associations have been observed between ON and anxiety, with research suggesting that major depressive disorder and anxiety disorders serve as risk factors for ON [10]. Strahler et al. [12] documented lower levels of well-being, life satisfaction, and higher stress levels among 713 individuals with ON (median age: 25 years) compared to those without the disorder. Furthermore, in a cross-sectional study involving 248 participants from Turkey (mean age: 42.6  ±  6.3 years), Yilmaz and Dundar [14] identified a notable prevalence of ON in individuals exhibiting elevated anxiety scores. The study’s findings also indicated a positive correlation between anxiety levels and the degree of obsession with healthy eating, thereby increasing the likelihood of orthorexic tendencies. Previous investigations in both males and females have additionally highlighted elevated ON levels among patients with somatoform disorders compared to healthy controls [15,16]. These collective findings suggest that individuals with orthorexic inclinations may adopt their dietary habits as a means of coping with mild manifestations of illness anxiety, thus implying a potential psychiatric comorbidity between ON and anxiety.

Looking at qualitative data and what people themselves have shared, a study involving nine participants (six females and three males) between the ages of 23 and 61 years old from the UK and USA revealed insights into the experiences of those self-reporting an obsession with healthy eating. Participants described feeling stressed due to their rigid food routine and need to control food choices, with holidays being highlighted as a key source of anxiety [17]. Participants also mentioned that as their stress levels increased, they felt a greater need to exert control over their eating habits. Individuals with ON often dedicate a considerable amount of time to researching food options, planning and organizing meals, and structuring their lives around their dietary preferences [11]. Additional qualitative findings emerged from two separate studies: one involving 141 participants over the age of 18 residing in the UK [18], and the other comprising 15 female bloggers aged 19–32 residing in the USA, England, Australia, and India [19]. Individuals exhibiting ON tendencies revealed distressing emotions, including fear and anxiety [18,19]. They described a vicious cycle of anxiety around food consumption, wherein heightened convictions that specific foods were threats exacerbated their digestive issues and anxiety. Nonetheless, the specific triggering factors and the beliefs surrounding food threats have not been fully investigated, highlighting the need for further research in this area.

The proliferation of discussions surrounding food, encompassing its benefits and risks, has led to the emergence of individuals who are informed yet anxious about health due to the influx of contradictory information [20,21]. In this context, health is perceived as both a right and a responsibility, compelling individuals to make informed choices. Amidst the abundance of sometimes conflicting information, the persistent pursuit of accurate knowledge regarding ingredients and expert advice contributes to feelings of anxiety and distress [22]. This pursuit of knowledge and anxiety over food reflect the concept of the orthorexic society introduced by Nicolosi [23]. Nicolosi contends that the increasing distance between producers and consumers has heightened the fears people develop around diet. According to Nicolosi, consumers become increasingly distressed as nowadays the course of food production (e.g., use of hormones), processing (e.g., use of preservatives), and preparation (e.g., cooking methods in the fast-food industry) remain opaque. Further, Nicolosi suggests that food has lost its communal and sociocultural meanings, while the human body has been framed as an individual project, thus encouraging personal agency. All these shifts in the sociocultural meaning of food, along with the capitalistic ways of offering food to consumers through open trade and less transparent processes, have heightened the stress around food that people are experiencing.

The field of ON has garnered considerable scholarly attention. However, there remains a dearth of qualitative inquiries into the experiences of individuals self-identifying with a fixation on healthy eating and exhibiting distress around food quality. Moreover, while the existing literature has established associations between ON and comorbid anxiety disorders, there is a notable absence of research exploring the specific triggers of anxiety in individuals with ON and the strategies they have developed to cope with stressful feelings. This represents a significant limitation in the current understanding of ON and underscores the importance of our study in addressing this gap in knowledge. Hence, our study sought to explore the triggers and circumstances that provoke anxiety-inducing beliefs and feelings in individuals who self-identify as concerned about healthy eating, along with the coping mechanisms devised to navigate these distressing feelings.

## 2. Materials and Methods

To develop a comprehensive understanding of the anxiety-provoking situations experienced by individuals with a preoccupation with healthy eating, we initiated a qualitative study utilizing a phenomenological approach. The study aimed to explore the following research questions: (1) What factors trigger anxiety in individuals with ON? and (2) What strategies have they developed for coping with these anxiety-inducing factors? In-depth, one-to one interviews were selected as the preferred method due to their ability to delve deeply into participants’ subjective experiences [24]. The interview script was developed to explore a range of topics related to participants’ dietary habits, emotions, thoughts, triggers, difficulties, motivations, and adherence to strict eating regimes. These interviews were conducted online via Teams, allowing for flexible and in-depth discussions with participants.

### 2.1. Participants

Given the absence of a universally accepted definition for ON and the lack of a standardized diagnostic framework, the recruitment of individuals with ON has inherent challenges. For these reasons, we opted to recruit participants who self-reported an obsession with healthy eating, which has been identified as a characteristic feature of ON [11]. Individuals were also required to meet the following criteria: (1) being over 18 years of age, (2) self-reporting experiencing anxiety related to the quality of their food, (3) being Greek, and (4) not having been diagnosed with any ED or undergoing treatment. These criteria were chosen to ensure the relevance of participants’ experiences to the research focus while minimizing confounding factors. The researchers posted an advertisement for the study on their social media accounts, and potential participants could express their interest by reaching out to them. Additionally, snowball sampling was employed to complement recruitment efforts. This method, commonly used in qualitative research, allowed for the inclusion of participants who may not have been reached through the initial social media advertisement. In total, 13 individuals (see Table 1) agreed to participate in one-to-one interviews. The participants in our study indicated that they began their preoccupation with healthy eating at various points in time, ranging from 3 months to 14 months. An informed consent was obtained prior to data collection, and respondents were carefully screened based on the aforementioned inclusion criteria to ensure compliance with ethical standards and the integrity of the study. After confirming their participation, interview appointments were arranged. Interviews, lasting approximately 30–45 min each, were recorded using an audio device to ensure accurate data capture.

### 2.2. Data Analysis

This study used thematic analysis [25]. The transcribed data underwent multiple readings, with recordings listened to several times to ensure transcription accuracy. This iterative process of ‘repeated reading’ and immersion in the data fosters researcher familiarity with the material [25]. Subsequently, the coding phase builts upon notes and insights derived from transcription and data immersion. Initial coding identified data features deemed relevant to the research questions. Equal attention was given to the entire dataset to capture recurring patterns comprehensively. The next stage involved theme identification, where codes that seemed similar or referred to the same aspect were grouped together. Themes lacking sufficient data support or exhibiting excessive diversity were discarded. Once a clear understanding of the themes and their interrelations was established, analysis progressed to defining and naming each theme. Each theme was meticulously defined and analyzed, considering its narrative within individual themes and its contribution to the overall dataset. Furthermore, concise yet descriptive names were crafted to succinctly capture the essence of each theme. The final stage involved selecting transcript excerpts to exemplify theme elements. These extracts effectively illustrated theme issues and provided clear examples of the points being addressed.

### 2.3. Rigor and Trustworthiness

To ensure the reliability of the results, detailed records were maintained throughout the analysis process, encompassing interview transcripts, field notes, and documentation related to coding preparation. Furthermore, member checking was conducted by inviting participants to review their transcripts for accuracy. Credibility was established through independent coding by both authors, followed by consensus meetings to review identified themes and discuss diverse interpretations until agreement on the findings was reached. Additionally, the results were cross-checked against the existing literature and studies.

Confirmability was reinforced by the inclusion of pertinent quotes to exemplify the emergent domains. Finally, this study adhered to the reporting criteria outlined in the ‘Consolidated criteria for reporting qualitative research’ (COREQ) checklist.

### 2.4. Research Team and Reflexivity

The interviews were conducted by the first author, a qualitative researcher with an interest in eating behaviors. Data analysis was primarily performed by the first author, with some transcripts also reviewed by the second author, an experienced researcher. Throughout the study, we engaged in ongoing reflexivity, documenting our reflections and challenging any preconceived notions about ON and coping strategies. By openly acknowledging our biases and actively working to set them aside during data collection and analysis, we aimed to enhance the objectivity and trustworthiness of our findings. Peer debriefing sessions were also instrumental in facilitating critical discussions about our interpretations and decisions, further contributing to the rigor of the research process. None of the recruited participants were acquainted with the interviewer. Prior to conducting the interviews, the interviewer established rapport with each participant, discussing the study objectives, research purpose, data reporting methods, privacy considerations regarding personal data processing, and other pertinent information.

### 2.5. Ethical Issues

The participants’ collaboration was voluntary, and each participant was informed about their right to omit questions or withdraw from the study without providing any reason. Prior to their participation, written informed consent was sought from each participant. Approval for the project was granted by the ethics committee at the researchers’ university.

## 3. Results

**Figure 1:**
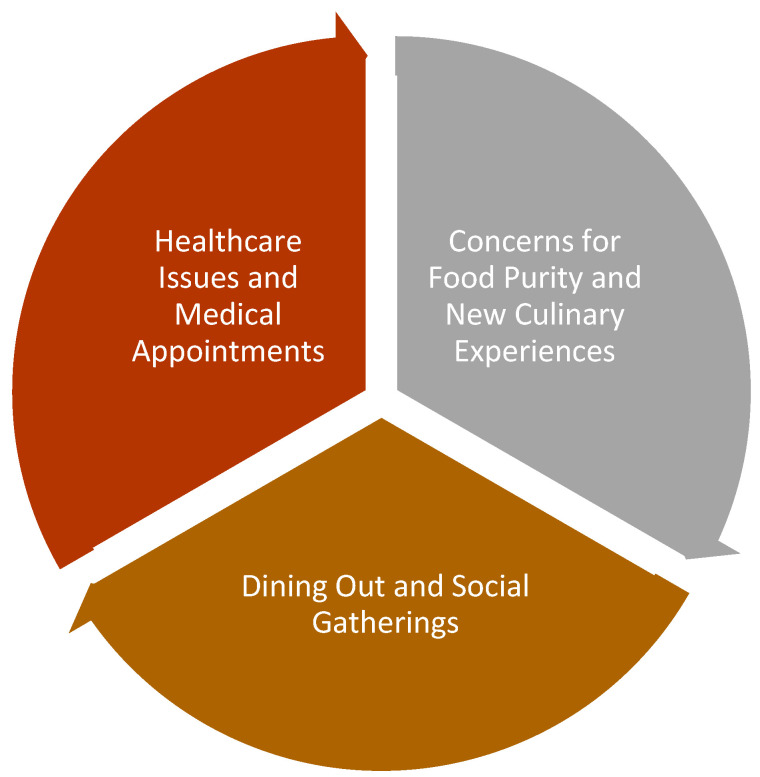
Themes identified from the interviews.

Analysis of the data identified three main themes, which can be seen in Figure 1. Each of the themes is discussed below, drawing on the participants’ accounts:

### 3.1. Concerns for Food Purity and New Culinary Experiences

All participants articulated apprehensions regarding the quality of the food and disclosed experiencing stress when faced with the task of sourcing ‘*high-quality*’ food products. According to the perspectives of the participants, food should be characterized by ‘*freshness*’, with detailed labeling of its provenance and ingredients. Many participants voiced concerns that a substantial portion of the food available for purchase contains ‘*additives*’ or ‘*enhancers*’, which compromise the nutritional integrity of the product. Furthermore, several participants expressed reservations about the hygienic standards of supermarkets, contending that these establishments may contribute to the contamination of fresh produce, particularly when products such as fruits or vegetables are left unpackaged. A subset of participants reported heightened anxiety regarding the safety of their food, citing concerns about potential contamination with ‘*insects*’ or even ‘*human flesh*’. Notably, these individuals recounted exposure to online discourses and documentaries portraying instances of meat sold in supermarkets and fast-food chains allegedly being adulterated with human tissue and insects. Such revelations instilled profound apprehension regarding the meat they consumed, leading some to abstain from meat products altogether:

*“I cannot eat meat anymore in my life. I watched a documentary that stated that meat is mixed with human flesh and insects. It disgusts me, and when I see people eating meat, I always feel stressed.”*

As a coping strategy, participants engaged in research to identify local supermarkets known for consistently offering fresh, high-quality food products and providing transparent information about the origins of their merchandise. While this approach provided comfort to participants by addressing their concerns about food provenance and alleviating fears of contamination, it also required them to invest time in locating suitable local supermarkets. In some cases, participants described the need to travel to different areas in order to purchase their food products:

*“I have to commute approximately 40 minutes by bus to reach the local shop where I buy all my food. Sometimes, I even have to purchase food for the entire week in one go, as it consumes a significant amount of time from my daily life. However, without making this effort, I wouldn* *’t have access to food.”*

Exploring new food products was likewise perceived as an anxiety-inducing endeavor, given the unfamiliarity with both the nutritional content and the perceived health effects, thus eliciting feelings of anxiety. Consequently, several individuals reported adhering to a monotonous dietary regimen as a coping strategy, consuming identical meals daily as a means of avoiding the anxiety associated with trying new foods and navigating limited options. Notably, one participant expressed apprehension regarding the repetitive nature of their meals and the perceived scarcity of viable food choices, consequently experiencing anxiety over the prospect of inadequate sustenance:

*“I feel anxious that there might not be any food suitable for me, or that there may be very limited options. Seeing others eat different things than me makes me want to stay away from social events if not necessary.”*

### 3.2. Dining out and Social Gatherings

Eating meals outside of their home, where participants lack control over the cooking process and the ingredients used, was identified as a significant source of anxiety. Weekends emerged as particularly challenging and anxiety-inducing periods, as they are commonly characterized by social invitations from friends and family to dine out. One participant described the anxiety-inducing nature of receiving such invitations, recounting palpitations and the desire to lie and fabricate excuses to decline. Restaurants and fast-food chains were identified as environments that exacerbated anxiety surrounding food consumption. Some participants expressed concerns about the potential for encountering ‘*stale*’ or ‘*contaminated*’ food, contributing to fears of foodborne illness or allergic reactions. This perception was rooted in the belief that restaurants and fast-food chains prioritize cost-cutting measures over investing in high-quality ingredients:

*“Whenever I go out to eat, I spend ages scouring menus to find something safe for me to eat. But it* *’s like restaurants don* *’t really care about what goes into their dishes. All they seem to care about is making a quick buck, so they end up serving cheap, dodgy food that could be full of God knows what.”*

Social gatherings involving food were consistently identified as anxiety-provoking situations. Participants expressed discomfort when faced with the prospect of consuming different foods from their peers and colleagues, citing it as a significant trigger for anxiety. This was particularly challenging when they struggled to find suitable dietary options in social settings, leading to feelings of discomfort, shyness, and fear of being judged. Consequently, many participants recounted occasions where they felt compelled to withdraw from social engagements to sidestep these uncomfortable situations. Participants perceived this approach as alleviating feelings of anxiety arising from a lack of control over food served outside the home or potential judgment from others due to their selective dietary choices. By self-isolating, some participants believed they could minimize deviations from their established dietary plans and avoid negative feelings of judgment:

*“Slightly larger social gatherings, such as office parties, can trigger anxiety for me, particularly regarding eating in front of others and fearing judgment of my eating habits. As an extreme introvert, any situation with a large number of people nearby makes me anxious, especially when dining out. Additionally, if I were the only one abstaining from meat or processed food due to my diet, I would feel particularly anxious. So, I just avoid every situation like this.”*

### 3.3. Healthcare Issues and Medical Appointments

The prospect of confronting a health issue or exacerbating existing health conditions was described as a source of significant stress. The majority of the participants expressed a preference for adopting clean or raw food diets driven by concerns surrounding health. These individuals articulated apprehensions about potential serious health ramifications, viewing clean eating as the primary means of mitigating such risks. Disclosure of pre-existing health conditions, including irritable bowel syndrome and coeliac disease, was common among participants. Furthermore, one participant, contending with high cholesterol levels, expressed persistent anxieties regarding the consumption of ‘unhealthy’ foods, fearing potential exacerbation of their condition and heightened risk of cardiovascular ailments such as heart disease or stroke. Engaging in food journaling throughout the day emerged as a coping strategy to monitor and assess the perceived ‘purity’ of consumed foods. Some participants found that maintaining a detailed food journal and reviewing it at the end of the day helped alleviate stress. This practice was viewed by participants as allowing them to evaluate their adherence to their food regimen and provided a sense of control, especially when their eating throughout the day had gone as planned:

*“I find food journaling quite helpful…At the end of the day, you can check what you have eaten and make sure that you have consumed all the right food for your body and health.”*

Along with healthcare concerns, medical appointments were considered to be stressful, as the results of the tests would indicate whether individuals had successfully adhered to their diet or needed to make stricter amendments. Some participants stated that they would feel ‘*devastated*’ if their tests came back negative or if their doctor advised caution. Medical appointments were considered indicators of whether stricter dietary control was necessary. To manage these stressful feelings, participants shared that they follow a strict dietary plan. Particularly before medical appointments, this plan might become stricter, involving cutting out more foods and ensuring the quality of their diet to increase the likelihood of positive results:

*“If my blood tests come back with high results…or if my doctor advises caution, I will feel devastated and stressed. I* *‘ll worry about the need for stricter dietary control and fear developing a medical issue…Before each appointment, I tighten my dietary regimen, cutting out more foods and ensuring quality…hoping for positive outcomes.”*

## 4. Discussion

We started this article with the premise that research within the field of ON is ongoing and has identified anxious thoughts among individuals with ON. However, specific circumstances and triggering factors that may induce anxiety in people are often neglected. Therefore, further exploration of anxiety and fear as emotions is warranted. This article contributes to the existing literature by identifying everyday situations that trigger anxiety in individuals with ON and examining the coping strategies they employ to manage these stressors and navigate overwhelming emotions.

### 4.1. Health Challenges and Food Neophobia

The majority of the participants in this study did not report any physical health challenge but four participants reported health issues such as irritable bowel syndrome, coeliac disease, and high cholesterol. The findings of this study revealed that participants, whether dealing with a health condition or not, adopted extreme forms of healthy eating practices not only as a means of disease prevention but also as a strategy for coping with and managing symptoms of health issues. This finding is consistent with the existing literature, which suggests that the primary motivation behind this fixation on healthy eating commonly arises from the desire to manage existing physical health concerns, prevent potential illnesses, and enhance overall well-being [1,2,3]. As acknowledged in our study, for these individuals, receiving a diagnosis of a health problem or being informed of its exacerbation could be seen as frightening prospects. Consequently, healthy eating was viewed in our study as possessing medicinal properties and played a crucial role in addressing stress and health concerns. These results align with previous research indicating a belief in food as a form of medicine among individuals with ON [17,18,19,20,21,22,23,24,25,26]. For these people, healthy eating may be viewed as more than just a means to maintain physical health or prevent diseases. Instead, they might perceive it as a form of self-medication, assuming that adhering strictly to a healthy diet can help alleviate stress and manage their health concerns.

Individuals with ON are known to exhibit a strong commitment to their dietary regimen. However, when they deviate from it, feelings of guilt and a desire for self-punishment, such as adopting a stricter diet or entering a starvation mode, may arise [1,2,3,4,5,6,7,8,9,10,11,12,13,14,15,16,17,18,19,20,21,22,23,24,25,26,27]. In our study, participants classified medical appointments and test results as stressful due to their potential implications for the success of their self-imposed dietary rules in maintaining good health, or whether stricter rules and greater self-discipline are warranted. In this context, medical appointments and test results were perceived as indicators of a successful commitment to the diet, prompting individuals to ‘punish’ themselves through stricter dietary measures. Manifestations of self-punishment, such as cleansing fasts, as proposed by Bratman [1,2,3,4,5,6,7,8,9,10,11,12,13,14,15,16,17,18,19,20,21,22,23,24,25,26,27,28], are considered key features of individuals’ behavior, alongside food restriction and food-related obsessiveness. Interestingly, discourses of self-punishment through restrictive diets have been found on pro-eating disorder websites [29], presumably indicating a similar method of managing preoccupation with both the quality and quantity of food seen in ON and other EDs. Given the health concerns involved in ON, more hazardous and problematic behaviors may manifest as coping mechanisms. As individuals in the study by Cheshire et al. [17] have shared, the higher the stress levels, the stronger the desire to exert control over their diet. Therefore, the greater the experienced concern and anxiety in everyday circumstances, the stricter the elimination of food and self-punishment can become.

An important finding in this study, which challenges the existing literature, is the identification of anxiety and fear surrounding the consumption of new foods, reflecting experiences of food neophobia. Food neophobia is characterized by an aversion to trying unfamiliar foods, and in severe cases, it can lead to malnutrition, limited social interactions, and psychological distress [30]. While previous quantitative studies exploring the relationship between ON and neophobia found no significant correlation [31,32], our study participants described how anxiety induced by unfamiliarity with the nutritional content and health implications of new foods led many of them to avoid trying such foods. Consequently, they adhered to a monotonous dietary regimen consisting of only familiar foods. It is important to note that this finding does not suggest that all individuals with ON exhibit food neophobia, but it may be a common characteristic among some individuals, driving them to restrict their food choices and experience heightened levels of anxiety and fear.

### 4.2. Food Purity and Stressful Beliefs

The current body of literature highlights the increased concerns regarding the quality of food exhibited by individuals with ON, which contrasts with the emphasis on food quantity typically associated with anorexia [8,9,10,11]. Building upon this existing research framework, our study further corroborates these findings by elucidating the pronounced focus on food quality among individuals with ON. Participants uniformly articulated the paramount importance of food quality, often expressing heightened levels of anxiety stemming from the challenges associated with sourcing ’high-quality’ food products. In accordance with prior research indicating that individuals with ON devote considerable time to researching food options and structuring their daily schedules to accommodate their dietary preferences [11], participants in our study reported spending a significant amount of time exploring food alternatives and seeking out markets that offer high-quality food. All participants expressed concerns about food purity, often mentioning challenges related to the lack of transparent information regarding additives and the origin of food items. Consequently, participants reported constructing more extreme beliefs regarding food contamination, foodborne illnesses, and allergic reactions. Importantly, some participants attributed their fearful beliefs about food contamination to exposure to documentaries and online platforms where discussions about food purity were prevalent. While healthy eating websites can provide valuable information about food, they can also exacerbate fears about food quality and promote extreme dietary practices [29]. This highlights the need for the critical evaluation of online sources and the importance of balanced information dissemination. Addressing misinformation and promoting an understanding of food quality could help individuals make informed dietary choices without succumbing to undue anxiety or extreme behaviors.

The fearful beliefs articulated by some participants in this study resonate with Nicolosi’s conceptualization of the orthorexic society. Our findings suggest that these apprehensions are cultivated in response to a perceived dearth of information regarding food ingredients and origins, a phenomenon elucidated by Nicolosi [23] as emblematic of the growing disconnect between producers and consumers—a pivotal factor in the emergence of food-related anxieties. Moreover, the proliferation of misinformation and unsubstantiated health claims serves to exacerbate apprehensions surrounding the consumption of purportedly ’toxic’ foods, thereby perpetuating a cycle of restrictive dietary behaviors [21]. Individuals with ON often deal with overwhelming anxiety in relation to the perceived healthfulness of their dietary choices, leading to a pronounced fixation on food quality that can significantly impinge upon both their physical and mental well-being [33]. Indeed, as our study delineates, these fearful beliefs engender a heightened sense of unease in conventional food procurement settings such as supermarkets and restaurants. In response, individuals may adopt coping strategies such as patronizing local supermarkets with transparent sourcing practices, eschewing such establishments altogether, or adhering to a monotonous dietary regimen comprised solely of ‘safe’ foods. The impact of ON on individuals’ daily functioning is multifaceted. The extremity of beliefs that can be developed as a result of the focus on a ‘pure’ diet and the limited information about the origins or the production of food can profoundly influence various aspects of their lives, including their mental well-being, and overall quality of life.

### 4.3. Self-Imposed Isolation

ON has been proposed to exert significant social ramifications on individuals’ lives [2]. Prior research characterizes individuals with ON as fostering a sense of superiority towards others regarding their dietary habits, driven by an obsessive pursuit of purity and adherence to self-imposed dietary rules [2,3,4,5,6,7,8,9]. This mindset may engender a perception of others as lacking in self-discipline, thereby fostering condescension towards those who do not adhere to similar dietary practices. However, our study yielded contrasting insights, with participants expressing feelings of discomfort, shame, and apprehension about potential judgment during social occasions. These sentiments stemmed from the perceived inability to make food choices that align with their dietary restrictions in social settings involving food consumption. Consequently, participants recounted instances of self-imposed isolation and avoidance of social interactions as coping mechanisms. These findings align with prior research that underscores the social isolation encountered by individuals with ON [1]. This isolation arises from a mismatch in dietary preferences with their social environment and the necessity to uphold control over their food selections across different social situations. Nonetheless, the attempt to avoid such discomforting feelings can constitute an alarming tendency at the expense of their social relationships and previously enjoyed activities.

The existing body of research has highlighted the significance of maintaining strict control over dietary habits among individuals with ON [11,12,13,14,15,16,17]. This entails a meticulous process of sourcing ingredients, planning meals, and organizing daily routines around dietary restrictions. Notably, our study identified weekends as particularly challenging periods for individuals with ON. During this time, social interactions often involve gatherings with friends and outings, leading to an influx of food-related invitations. Previous qualitative research has shown that holidays also trigger anxiety among individuals with ON [17], suggesting that both weekends and holidays present challenging situations. This is likely due to the unstructured nature of these periods, which contradicts the structured routine desired by individuals with ON to maintain control over their diet. Consequently, dining outside the home and attending social events were perceived as anxiety-inducing situations due to the lack of control over ingredient selection and food preparation. In response to such scenarios, one participant recounted resorting to fabricating excuses to decline invitations, thereby maintaining control over their dietary regimen. This finding highlights the extent to which individuals with ON may deliberately opt for self-isolation to uphold their dietary principles. Further, it emphasizes the dedication to self-imposed rules and the lengths to which individuals are willing to go to uphold a sense of ‘security’ and commitment to their dietary ethos.

### 4.4. Limitations and Future Research

The primary limitation of this study pertains to the challenge of generalizing the findings, inherent in the qualitative methodology employed. Despite our efforts to recruit a larger sample, qualitative research often involves working with smaller sample sizes when exploring complex phenomena like ON. However, while the sample size may appear limited, it falls within the recommended number of interviews necessary to achieve data saturation in qualitative research [34,35]. Data saturation appeared to have been reached for this study, and the comprehensive nature of the qualitative research provided rich insights into the investigated phenomenon. Nevertheless, we encourage future studies to delve deeper into this topic and include a larger sample size. Doing so would facilitate the generation of additional insights and enable comparisons with our findings.

Our study aimed to recruit individuals from diverse age demographics, but the participants included were primarily in their 20s and 30s. This demographic trend may suggest that individuals within this age range are more active on social media or more inclined to share their thoughts and experiences regarding their diet. However, future research endeavors should strive to investigate fearful thoughts and triggering factors among more diverse age demographics. This would enable a thorough examination of whether similar or different fearful and anxiety-provoking situations are encountered across different age groups considering that presentations of ON have been found in both younger and older populations [5,6,7,8,9,10,11,12,13,14,15,16,17,18,19,20,21,22,23,24,25,26,27,28,29,30,31,32,33,34,35,36]. An additional limitation in this study is the inclusion of participants from a specific nationality, potentially constraining the understanding of stress-inducing situations experienced by individuals with ON across different cultural contexts. Future research endeavors should aim to investigate these phenomena across diverse nationalities, as contextual factors between countries may yield valuable insights. Finally, this study revealed a commonality among individuals practicing healthy eating for varying durations. Participants presented overlapping fearful beliefs and coping strategies. Notably, individuals with ON may harbor extreme beliefs regarding food contamination, leading them to engage in behaviors like avoiding social gatherings and certain locations or restricting their food intake to maintain a sense of safety. Future research could further explore these beliefs and investigate to what extent individuals adhering to longer periods of a dietary regimen uphold different beliefs and imposed restrictions.

## 5. Conclusions

In conclusion, this study sheds light on the triggering factors of anxiety and fear experienced by individuals with ON and the coping strategies they employ to manage these distressing emotions. By identifying everyday situations that provoke anxiety and fear, such as health concerns, medical appointments, encounters with new foods, and social events involving food, this study contributes to a deeper understanding of the challenges faced by individuals with ON in their daily lives. Moreover, this study underscores the intricate nature of navigating through these situations and the strategies, such as self-isolation or repetitive food choices, that individuals adopt to manage these distressing thoughts. These findings have significant implications for future researchers, clinicians, and policy makers. Firstly, future research endeavors should aim to delve deeper into the social factors contributing to anxiety and fear among individuals with ON. Understanding the underlying mechanisms driving these emotions can inform the development of targeted interventions aimed at mitigating distress and improving overall well-being. Secondly, clinicians working with individuals with ON can benefit from the insights provided by this study. By recognizing the pivotal events that trigger anxiety and fear, clinicians can tailor treatment approaches to address these specific challenges. Additionally, understanding the coping strategies employed by individuals with ON can inform the development of more effective therapeutic interventions aimed at promoting adaptive coping mechanisms and reducing reliance on maladaptive behaviors. Lastly, policy makers have a role to play in addressing the broader societal factors that contribute to the development and perpetuation of orthorexic behaviors. By promoting food literacy and transparency in the food industry, policy makers can empower consumers to make informed choices about their dietary habits. For instance, policy makers can implement regulations that require transparency in food production and clear and accurate labeling of food products, including information about provenance, additives, preservatives, and nutritional content. Such measures can help alleviate anxiety about food quality, enabling individuals to make more confident and informed choices regarding their dietary habits. In sum, this study underscores the importance of addressing anxiety and fear in individuals with ON, and highlights the need for comprehensive approaches that address the complex interplay of individual, social, and environmental factors.

## Figures and Tables

**Table 1:** Participants’ characteristics.

Code	Sex	Age (Years)	Health Issue
P1	Woman	23	None
P2	Woman	32	None
P3	Man	22	None
P4	Man	28	None
P5	Woman	21	Irritable Bowel Syndrome
P6	Woman	27	None
P7	Woman	24	Coeliac Disease
P8	Woman	26	None
P9	Woman	26	Irritable Bowel Syndrome
P10	Man	36	None
P11	Woman	25	None
P12	Man	38	Cholesterol
P13	Man	23	None

## Data Availability

Data are available upon reasonable request.
